# Role of Alpha-Band Oscillations in Spatial Updating across Whole Body Motion

**DOI:** 10.3389/fpsyg.2016.00671

**Published:** 2016-05-06

**Authors:** Tjerk P. Gutteling, W. P. Medendorp

**Affiliations:** Donders Institute for Brain, Cognition and Behaviour, Radboud UniversityNijmegen, Netherlands

**Keywords:** parietal cortex, oscillations, visual stability, self-motion, vestibular, electroencephalography

## Abstract

When moving around in the world, we have to keep track of important locations in our surroundings. In this process, called spatial updating, we must estimate our body motion and correct representations of memorized spatial locations in accordance with this motion. While the behavioral characteristics of spatial updating across whole body motion have been studied in detail, its neural implementation lacks detailed study. Here we use electroencephalography (EEG) to distinguish various spectral components of this process. Subjects gazed at a central body-fixed point in otherwise complete darkness, while a target was briefly flashed, either left or right from this point. Subjects had to remember the location of this target as either moving along with the body or remaining fixed in the world while being translated sideways on a passive motion platform. After the motion, subjects had to indicate the remembered target location in the instructed reference frame using a mouse response. While the body motion, as detected by the vestibular system, should not affect the representation of body-fixed targets, it should interact with the representation of a world-centered target to update its location relative to the body. We show that the initial presentation of the visual target induced a reduction of alpha band power in contralateral parieto-occipital areas, which evolved to a sustained increase during the subsequent memory period. Motion of the body led to a reduction of alpha band power in central parietal areas extending to lateral parieto-temporal areas, irrespective of whether the targets had to be memorized relative to world or body. When updating a world-fixed target, its internal representation shifts hemispheres, only when subjects’ behavioral responses suggested an update across the body midline. Our results suggest that parietal cortex is involved in both self-motion estimation and the selective application of this motion information to maintaining target locations as fixed in the world or fixed to the body.

## Introduction

In daily life, we constantly move our body around. During this motion, we seem to have no difficulty in keeping track of important objects, even when they disappear from view.

To track objects accurately, it is important that the brain knows whether the object is fixed relative to world or moves with the body (Baker et al., 2003). For objects that remain fixed in the world, the brain is known to make use of spatial updating mechanisms that adjust locations for the intervening motion. However, when the object moves with the body, the brain should ignore the motion, and maintain the location of the object as initially stored in memory.

While the behavioral outcomes of spatial updating across body motion have been studied in detail [see Klier and Angelaki (2008) and Medendorp (2011), for reviews], the underlying neural components, and their interactions, are now beginning to be understood. The objective of the present study is to understand the role of cortical oscillations in spatial updating across whole body motion using electroencephalography (EEG).

Recently, we performed the first study in this domain (Gutteling et al., 2015). Under continuous recording of EEG, we passively translated human subjects while their gaze was fixed to a world-fixed visual point. Prior to the motion, a target was briefly presented, whose world-fixed location had to be remembered during the motion. We found that the internal representation of this target is reflected by alpha band modulations in scalp parieto-occipital areas, which are remapped *trans*-hemispherically when the remembered location reverses sides relative to subject’s gaze direction during the motion. This suggests that the brain encodes and updates world-fixed target locations in egocentric, gaze-based coordinates during whole body motion, extending previous findings of head-fixed saccadic updating (Duhamel et al., 1992; Colby and Goldberg, 1999; Medendorp et al., 2003; Merriam et al., 2003).

It is important to note, however, that the motion in this study consisted of combined body and eye motion. This means that we could not distinguish whether the update was based on vestibular signals only, the accompanying gaze signals, or a combination of the two. We also did not compare conditions that required updating and those that did not.

In the present study, we therefore test the role of oscillatory activity in spatial updating during whole body motion, based on signals from the vestibular system only. By using either body- or world-fixed targets during whole body motion with constant, central gaze, we aim to isolate activity related to the coding of initial target presentation, its storage in memory, vestibular motion processing, and spatial updating processes.

Previous literature has suggested a role of alpha band oscillations in the coding of all these components. More specifically, alpha band activity in parietal regions has been implicated in processing and memorizing visual stimuli (Jensen et al., 2002; Roux and Uhlhaas, 2014; Foster et al., 2016). During saccadic eye movements, alpha band activity has been shown to remap (Van Der Werf et al., 2013). Importantly, a recent study has also associated vestibular processing with a suppression of alpha power in bilateral temporo-parietal scalp regions (Gale et al., 2015), providing evidence that vestibular signals reach parietal cortex in humans.

The main goal of the present study was to investigate the role of alpha band activity in a task that requires the interaction of vestibular processing and target coding to update memorized visual targets during whole body motion. Based on previous work, we hypothesize that vestibular activity modulates alpha power in parietal and temporo-parietal areas, whereas target coding evokes lateralized alpha, consistent with a gaze-based coordinate system. Spatial updating will be seen as a *trans*-hemispheric shift of these representations, along with a parietal modulation, but only when the remembered target location reverses sides relative to the subject’s gaze.

## Materials and Methods

### Participants

Twenty-nine right-handed, healthy participants (mean age 26.7 years, range 18–61, 13 women), free of any neurological or vestibular disorder and with normal or corrected-to-normal vision gave their informed consent to participate in the experiment according to the local ethics committee. Five participants were excluded due to prescreen restrictions (undisclosed medication, one subject), failure to perform the task (three subjects, one showing excessive overestimation of motion, two showing failure to adhere to task instructions) or excessive head movement (one subject). Thus, the data used in the analyses included 24 participants (mean age 25.7 years, range 18–45, 11 women).

### Setup

**FIGURE 1:**
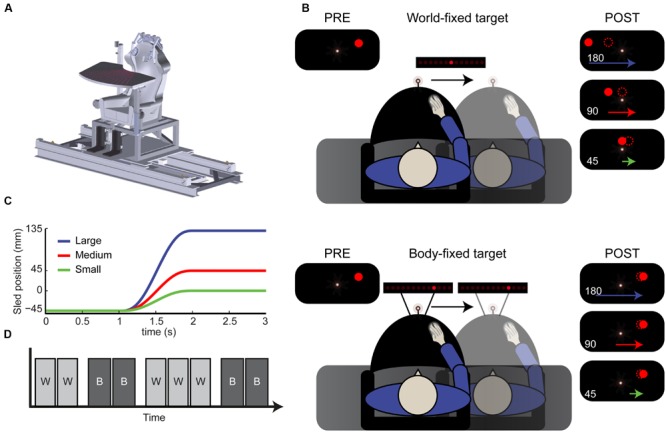
**Paradigm. (A)** Schematic drawing of the vestibular sled. **(B)** Top view of the experimental setup. Subjects performed a spatial updating task with world-fixed (top) and body-fixed (bottom) targets. Using a sled-mounted fixation, a target was flashed, resulting the percept shown in the top left inset. After displacement, either 180, 90, or 45 mm subjects indicated where the remembered target was (dotted circle). This was generally underestimated in the world-fixed condition (compare with filled dots, the actual location). **(C)** Motion profiles of the large (180 mm), medium (90) and small (45) displacements. **(D)** Example of condition block order. W, world-fixed; B, body-fixed. Conditions alternated every two or three blocks and started with either world or body-fixed targets.

We used the same setup as in Gutteling et al. (2015) and therefore only provide a brief description here. All measurements took place in a completely darkened room. Subjects were seated in a custom-made linear sled capable of translating sideways along an 800 mm track (**Figure 1A**). The subjects’ interaural axis was aligned with the sled motion axis. Participants were restrained with a five-point seat belt. Head motion was restrained with a modified over-ear headphone fixed to the sled. Responses were given by a computer mouse (see paradigm). During the task, white noise was presented using in-ear earphones, masking sound produced by sled motion. An infrared camera, which was mounted in the room and enabled a view of the participant in the dark, was used to check for excessive body- or head movements.

A 96-channel active electrode EEG system (Brain Products, Gilching, Germany) was attached to the sled. The over-ear headphone prohibited EEG recordings from electrodes located around the ears (FT9/10, T7/8, TP7/8, and TP9/10), leaving 88 active recording sensors. Horizontal and vertical electro-oculograms (EOGs) were also recorded. Impedance of all electrodes was kept below 20 kΩ, an adequate level for this active system. EEG and EOG signals were sampled at 1,000 Hz (amplifier bandwidth 0.016–1,000 Hz, internal sampling rate 5 kHz) and then saved to disk.

Eye movements were recorded using an EyeLink 1000 system (SR research, Kanata, ON, Canada), recording binocularly at 500 Hz with online head motion correction. The camera system was mounted on the sled and used infrared illumination outside the visible spectrum.

Visual stimuli were presented using red light-emitting diodes (LEDs). A single LED was attached to the sled at a distance of approximately 720 mm centrally in front of the subjects’ eyes. This LED served as a body-fixed fixation point. A 450-mm-wide array of LEDs, consisting of 180 square (2 mm × 2 mm) LEDs with a spatial separation of 2.5 mm (center to center), was used to present target and probe stimuli. These LEDs had a luminance <1 cd/m^2^. The array was oriented in parallel with the sled motion axis, and could either be mounted on a tripod on the floor where it was stationary, displaying world-fixed targets, or attached to the sled, displaying body-fixed targets (see paradigm). In both target conditions, the array was placed ∼860 mm (140 mm behind fixation) from the subjects’ eyes. The fixation point and LED array were displaced vertically by a few millimeters, such that they did not occlude each other and were approximately at eye level.

The experiment was controlled with custom software programmed in Python programming language. EEG and EyeLink data were recorded on separate computers and events were synchronized using triggers sent by the task computer.

### Paradigm

The task consisted of a spatial updating paradigm with world- and body-fixed targets, see **Figure 1B**. At the start of each trial, the body-fixed fixation turned on, which the subject had to fixate during the entire trial. After 1 s, a target was presented either to the left or right of the central fixation point for 100 ms. In the *world-fixed* condition the target was presented 3° (45 mm) ± 0.3° (5 mm) jitter, to the left or right of the fixation point. One second after target onset, subjects were passively translated sideways, with a displacement of either 45, 90 or 180 mm in the direction of the target using a minimum jerk profile (**Figure 1C**). As a result, the actual (invisible) target location ended up either straight ahead of the subject 0° (0 mm), 3° (45 mm), or 9° visual angle (135 mm) to the other side of fixation. After a 1 s post-motion delay, a random LED on the array turned on. Using the mouse, the horizontal position of this LED on the array could be controlled. Subjects had to shift this LED to the remembered location of the target, taking as much time as needed. Subjects performed the world-fixed condition in blocks of 54 trials.

The *body-fixed* condition was performed in the same way (**Figure 1B**, bottom), except that targets were presented at ten different locations relative to fixation (equally spaced between -9.2 and +9.2°). After the motion, the subject had to indicate the remembered location of the target in *body*-coordinates. Subjects performed the body-fixed condition in blocks of 60 trials. Thus, in total there were twelve conditions: two target types (world/body), three displacement sizes (45, 90, and 180 mm) and two displacement directions (left/right).

The subject’s task was thus to encode the initial target, update its memorized location over the course of the sled motion in either a body- or world-fixed manner, and indicate the internally updated location using a mouse. Displacement size was randomized within blocks. The body- and world-fixed conditions alternated in a block-wise fashion; this was clearly instructed before each block. The initial block was either world or body-fixed and was randomized over subjects. In total, subjects performed nine blocks (5 world-fixed, 270 trials; 4 body-fixed 240 trials, see **Figure 1D**). A block lasted approximately 5–6 min and the experiment ∼1 h in total including breaks.

### Analysis – Behavior

All analyses were performed using MATLAB (Mathworks, Natick, MA, USA). Mouse responses were used to calculate the mean remembered location and error (i.e., the difference between the actual and remembered target location) for every condition per subject. As a measure of updating performance independent of direction or displacement size, the updating gain (γ) was calculated as the proportional difference between the actual (*D*) and estimated (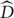) displacement size. As the displacement size is identical to the target displacement (from the subjects’ perspective), the estimated displacement 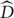 = D - error, and γ is calculated as: 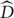/D, where the error is positive when making an underestimation of motion. This way, the gain factor is 1 when perfectly updating the target and 0 when no update is made.

### Analysis – Eye Movements

Eyelink data epochs were created using co-recorded EEG triggers and separated per condition. Normalized eye traces were added to the EEG dataset after ICA (see Analysis – EEG) and used for subsequent manual inspection.

### Analysis – EEG

Analyses of the EEG data were performed using the Fieldtrip toolbox (Oostenveld et al., 2011). First, EOG was separated and bad (extremely noisy, often exceeding 50 μV) channels, identified by visual inspection of the data, were removed before subjecting the data to ICA decomposition ‘runICA’ implementation, using the logistic infomax algorithm of Bell and Sejnowski (1995). Components with a clear ocular or muscular origin were removed, following artifact signatures as identified in McMenamin et al. (2010). After recomposing the data, filtering was applied (low-pass 140 Hz; high-pass 1 Hz; two-pass Butterworth 49–51 Hz band stop filter). EOG data was filtered (low-pass 20 Hz; high-pass 1 Hz) before being added to the data along with the eye movement data from the Eyelink eye tracker. Manual artifact rejection was performed in the time domain, removing trials with clear (incidental) artifacts, such as spikes and atypical muscle or ocular artifacts. Missing channels (removed before ICA) were interpolated using a distance-weighted nearest-neighbor approach. All data were re-referenced to average reference (Bertrand et al., 1985).

Time domain data were transformed to the frequency domain using Morlet wavelets (σ = 7, resulting in an average frequency resolution of 2.8 Hz and a 222 ms wavelet length in the 8–12 Hz alpha range) over a frequency range of 2–80 Hz. All trials were baselined using a pre-stimulus baseline (500–0 ms before target presentation). Data were sorted by condition (12) and saved as both single trial estimates and condition averages. As pointed out in the Introduction, the analyses focused on effects in the alpha band, in the 8–12 Hz range.

Contrasts were created to extract activity related to target coding, vestibular processing, and spatial updating. To isolate activity related to target coding, data from the body-fixed condition were used. Per subject, power in trials was averaged separately for trials where targets were presented either at the outer left or right locations. These averages were collapsed over displacement size and direction. This yielded two averages for left and right targets without updating, i.e., the target remained stationary relative to the observer.

Vestibular activity was isolated by contrasting large (L, 180 mm) and small (S, 45 mm) displacements in the body-fixed condition. EEG data from the rightward (r) motion was mirrored (^∗^) along the midline. As there were no significant differences between vestibular activity patterns for left- and rightward motion (dependent samples linear regression tests per time 0.5 s time window, all *p* > 0.08), the rightward motion data was averaged with the leftward (l) motion data: (Ll–Sl + Lr^∗^–Sr^∗^)/2.

Spatial updating activity was computed, which reflects the application of the vestibular motion signal in the update of the remembered location of a world-fixed target. Subjects’ trial-by-trial behavioral responses were used to select trials where subjects indicated that the remembered target shifted sides relative to fixation during the update, e.g., a target that was initially presented to the left of fixation was indicated as being right from fixation after the motion. Physically this was the case with medium and large displacements, but the percept depended on the subjects’ updating gain. Thus, the updated location of the remembered target, after motion, could either remain at the same side from the body midline and gaze (referred to as *within* updating), or be shifted to the opposite side from the midline/gaze (referred to as *across* updating).

We examined whether the quality of updating is correlated with power modulations in the alpha band, as suggested by our previous paper (Gutteling et al., 2015). For each subject the world-fixed gain value, based on the behavioral responses, was correlated with the difference in alpha band power before (*t* = 0–1 s) and after (*t* = 2–3 s) motion per electrode in the world-fixed condition. The pre–post motion alpha difference was chosen because it represents the alpha band fluctuation across motion in a single parameter. An independent samples linear regression was performed separately for each motion size and direction, because there were consistent differences in gain depending on the size of motion within subjects. Across subjects, the resulting beta-values were tested for significance, resulting in regression *t*-values, and corrected for multiple comparisons using Bonferroni correction.

## Results

We assessed the neural signatures of target coding, vestibular motion processing, and spatial updating using EEG. Subjects had to remember a briefly flashed world-fixed target (remaining stationary in the world) or a body-fixed target (moving with the subject) across a passive motion of various sizes and report its remembered location using a mouse-guided response.

**FIGURE 2:**
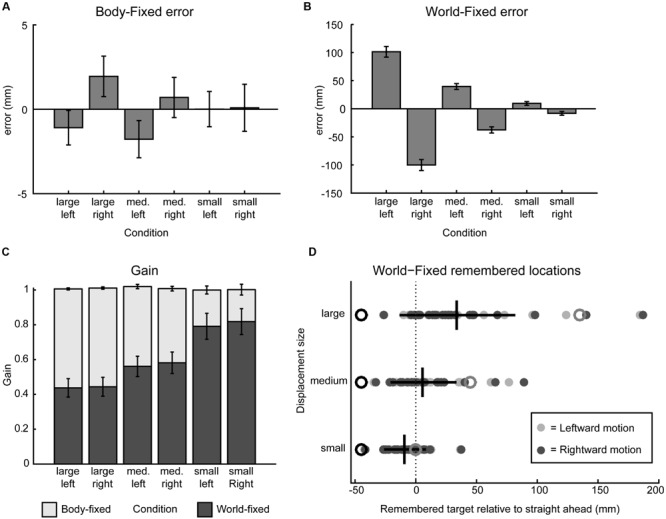
**Behavioral results. (A)** Signed error, calculated as the mean difference between actual and indicated target position, in the body-fixed condition for all displacement sizes and directions, pooled across participants. Negative error indicates error to the right, error bars denote standard error. **(B)** Same as A in the world-fixed condition. **(C)** World- and body-fixed behavior as expressed by gain factors per condition, where the darker bars indicate world-fixed gain and lighter the body-fixed gain. World-fixed gain factors increase with smaller motion. Error bars denote standard error. **(D)** Remembered target positions for the world-fixed condition, collapsed over left and right motion, relative to fixation (0, dashed line) for each participant (filled circles). Initial targets are indicated by black open circles, actual target locations after motion are indicated in gray open circles. Mean remembered locations do not cross fixation in the small motion condition, but do cross in the medium and large displacement conditions, as indicated by the vertical black lines (mean perceived locations after motion, horizontal lines denote standard deviation).

**Figure 2** shows the behavioral results. Updating errors of body-fixed targets (**Figure 2A**), which are calculated as the difference between the actual and remembered spatial location were small (<5 mm), and not significantly dependent on size (small/medium/large) and direction (left/right) of the displacement (ANOVA, all *p* > 0.12). However, in the world-fixed condition (**Figure 2B**), updating errors were substantial and show a significant effect of displacement direction [*F*(1,23) = 68.9, *p* < 0.001] and a significant size × direction interaction [*F*(2,22) = 91.4, *p* < 0.001]. While these errors depend on displacement size, as expected, these errors are not proportional to the displacement size, as reflected in a non-constant gain factor. The updating gain for world-fixed targets (**Figure 2C**), which was on average 0.61 (SD 0.30), was higher for small displacements (mean γ = 0.80) than medium (mean γ = 0.57) and large (mean γ = 0.44) displacements. A repeated measures ANOVA revealed that these differences were significant [*F*(2,22) = 63.9, *p* < 0.001]. *Post hoc* tests show that these differences are significant for all sizes (paired samples *t*-test, large vs. medium size *t* = -10.31, *p* < 0.001; medium vs. small *t* = -6.85, *p* < 0.001; large vs. small *t* = -9.24, *p* < 0.001). There were no other significant effects or interactions (all *p* > 0.38).

Following ideal geometry, for small displacements, an egocentric updating process should keep the remembered location of a world-fixed target at the same side of the body midline/gaze as where it was initially presented, whereas for larger displacements the updating process should cause this location to shift sides. On average, this was found: the small displacements show within updating and the medium and large displacements show across updating. However, because the updating gain is smaller than unity (the ideal gain) and shows considerable variability, there are a substantial number of trials that show within updating, and not across updating, for the medium and even large displacements (**Figure 2D**). The question is whether these behavioral observations in these trials can be associated to the spectral power modulation as detected in the EEG signals.

**FIGURE 3:**
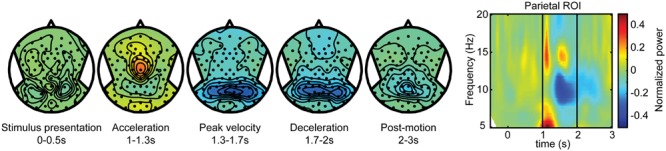
**Self-motion estimation activity.** Electroencephalography (EEG) scalp topoplots **(Left)** of alpha-band activity for time windows before, during and after motion for the self-motion contrast. This contrast was created by using body-fixed data, averaged over all target locations, and subtracting small motion from large motion. Rightward motion was flipped across the midline and averaged with the leftward motion. This isolates activity that increases with larger motion, while subtracting out effects of target coding and constant activity. **(Right)** Time-frequency plot for a parieto-occipital ROI (Pz, PO3/4, POz, and PPO1/2h). Motion occurs between *t* = 1 and 2 s.

As described in the Section “Materials and Methods,” we distinguished the alpha band power components of the electroencephalographic signals in terms of self-motion processing, target coding, and spatial updating processes. To isolate the self-motion processing we contrasted large and small displacements in the body-fixed condition. **Figure 3** shows scalp EEG topography of the power modulations of the alpha band for this contrast during five non-overlapping time intervals. Because target processing is subtracted out by this contrast, no significant topographic alpha band power distribution is seen during the first interval. The subsequent second to fourth intervals show the power changes during three phases of motion: acceleration, peak velocity, and deceleration. During the acceleration phase, there is an alpha band power increase over scalp central regions as well as an initial power reduction over central parietal electrodes, extending to temporal electrodes. This suppression is maximal during peak velocity and deceleration, and diminishes after motion ended (post-motion interval). The time-frequency plot, depicting activity from central parietal electrodes for a larger frequency band, shows that the power reduction is restricted to the alpha band while the initial increase is a more broadband response confined to motion onset.

**FIGURE 4:**
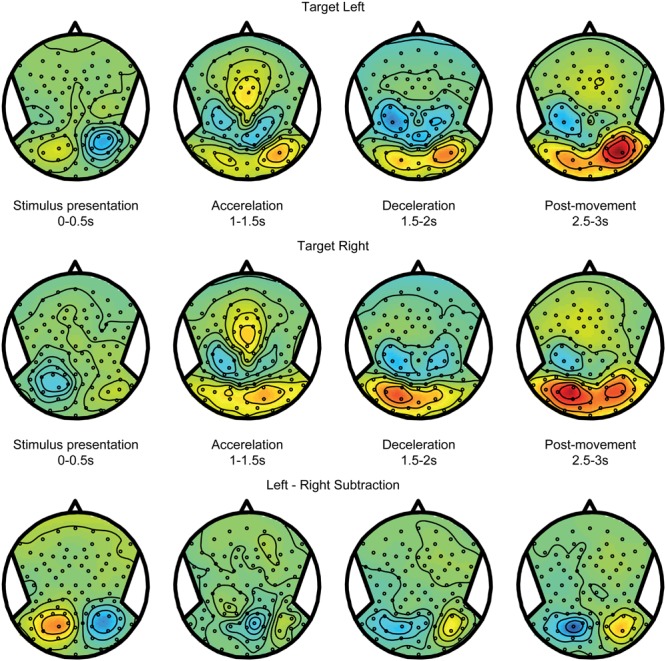
**Target coding activity.** EEG alpha-band scalp topoplots of the body-fixed condition, separately averaged for targets on the left **(Top)** and right **(Middle)**, before, during and after motion. Activity is averaged over left and right motion directions. **(Bottom)** Subtraction of left and right target conditions (above). This contrast isolates effects of left vs. right target.

**Figure 4** shows alpha power modulations related to encoding and maintaining a spatial target over time, without recruiting any spatial updating processes. To compute this component, data from the body-fixed condition were averaged together according to the location of the presented target relative to fixation (**Figure 4** top for left and middle for right presented targets). Because the target was body-fixed, no egocentric updating was required and targets had to be remembered at the same location relative to body during the motion. As shown, the brief visual presentation of the target evoked a reduction of alpha band power during the first 0–0.5 s interval, at electrodes covering the hemisphere contralateral to the target. While memorizing the target during the motion, this power reduction evolved into a strong increase. Subtracting the alpha band power values for leftward and rightward target location (bottom panel) reveals a clear lateralization that changes polarity over time. This suggest that, while the presentation of a visual target induces a lateralized alpha reduction, remembering its body-fixed location across motion is accompanied by lateralized alpha increases.

**FIGURE 5:**
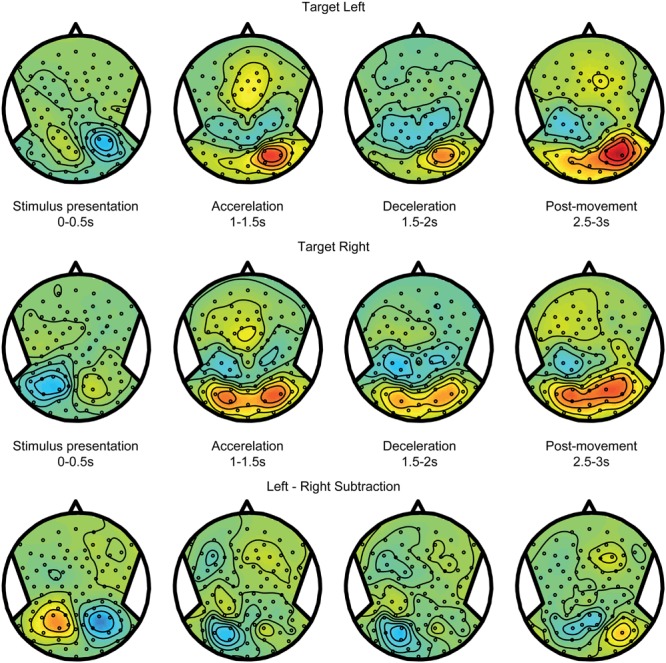
**Spatial updating within hemifields.** EEG topoplots from the world-fixed condition, averaged over trials where subjects indicate that the target remains on the same side as the initial target on the left **(Top)** and right **(Middle)**. Visually presented targets are initially reflected by contralateralized alpha reductions, and evolve into alpha increases contralateral to the remembered target after motion. **(Bottom)** Subtraction of left and right target conditions, isolating activity related the target representation.

Spatial updating processes are recruited in the world-fixed condition, during which the internal egocentric representation of the target must be updated to correct for the intervening motion. The updating errors (see **Figures 2B,D**) indicate whether targets were remembered after motion at the same side of the body midline as the initial location, or shifted to the opposite side. Using these responses, we sorted the trials in two categories: *within* updating trials and *across* updating trials (see Materials and Methods). We expect the activity pattern in within updating trials to be similar to the pattern in trials where targets shift with the body – the body-fixed condition. **Figure 5**, top and middle panels, show the averages for within updating for left and right target presentations in the alpha band. As can be seen, the initial visual presentation induces a lateralized alpha reduction in the hemisphere contralateral to target presentation. Over time, the alpha band power increases in the same hemisphere, showing a temporal evolution very similar to the body-fixed condition. This consistency is further clarified by comparing the power differences between left and right targets, showing a clear lateralization (see bottom panels **Figures 4** and **5**).

**FIGURE 6:**
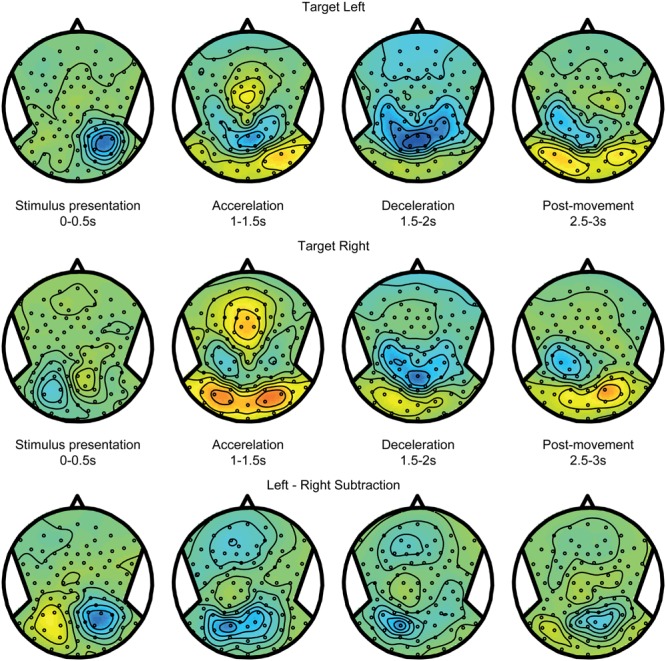
**Spatial updating across hemifields.** Same as **Figure 5**, but averaged over trials where the target is updated across hemifields, and is thus perceived to cross fixation. Consequently, the remembered target representation can be seen to remap to the other hemisphere after motion.

**Figure 6** shows the alpha band power modulations for the across updating trials. Initially, after target presentation, the power reduction is similar to the within updating trials (compare **Figure 5**). However, during the motion, it can now be observed that this power reduction turns into a power increase in the other hemisphere, which is the hemisphere that is contralateral to the updated location relative to the midline. Note that there is a non-specific increase in alpha band power in the right hemisphere, regardless of target location (see **Figures 4** and **5**). This difference between updating left and right presented targets is shown in the bottom panel of **Figure 6**.

**FIGURE 7:**
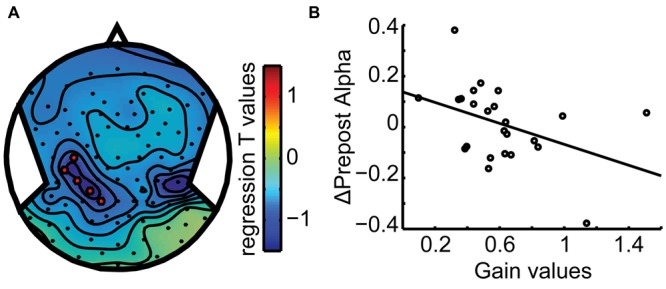
**Behavioral Correlation. (A)** Scalp topography plot of the regression *t*-value distribution for the gain-alpha pre–post analysis. A left parieto-temporal cluster (C3, CP6, CP3, P1, CCP5h, and CPP3h) shows a significant negative correlation **(B)** with gain values (*r* = -0.41). Data points mark subject averages of gain and pre–post alpha modulation over the three displacements. This indicates that higher performance (gain) goes along with a larger modulation of alpha band activity, specifically more alpha reduction after motion.

Taken together, **Figures 5** and **6** show that the dynamics of the alpha band power follows the behavioral observations: it remaps across hemispheres in the across updating trials and remains with the same hemisphere if subject perceive it to remain at the same side from the midline and gaze, similar to no updating in the body-fixed condition. To further assess whether the relationship of alpha band power and updating behavior, we correlated the power difference across the motion with the gain factor, separately for each subject. This yielded a significant correlation (*r* = -0.41, *p* = 0.046) for a left central parietal cluster, see **Figure 7**, indicating that higher gain values are associated with larger alpha reductions in this area.

## Discussion

We studied the role of alpha band oscillations in spatial updating across whole body motion. By presenting targets as either body- or world-fixed, we distinguished the oscillatory activity in components related to the coding and storage of the target, the processing of the body motion, and updating processes. Our results show that the processing of body motion is reflected by a power reduction over central parietal areas, whereas target coding is seen in power modulation over parieto-occipital areas, contralateral to its location relative to the body midline (and gaze). In the same regions, spatial updating processes remap the oscillatory activity across the two hemispheres if the body motion causes the target to shift sides. Thus, the parietal cortex is crucially involved in both the estimation of self-motion and spatial updating of remembered targets, consistent with its role as an integration area for visual and vestibular signals (Zhang and Britten, 2011; Gale et al., 2015; Gutteling et al., 2015).

Behavioral results show that subjects could well remember the locations of targets that move with the body, making only minor, non-significant errors. Subjects were also able to update world-fixed targets, despite the absence of active motion and vision. However, updating performance was not perfect, showing systematic errors, depending on the size and direction of the motion. The mean gain had a value of 0.61, which is consistent with our previous studies testing updating during passively induced body motion (Clemens et al., 2012; Gutteling et al., 2015). We found the updating gain of world-fixed target to reduce with the size of the body displacement. A possible explanation is that the body-fixed fixation point creates a strong stationary prior. It may be that there is signal dependent noise on the vestibular signal (Mallery et al., 2010), creating a noisier signal with larger displacements. The resultant estimate of the motion would then be more biased toward being stationary with larger displacements. While the average gain was below one for all three displacements, the variability of this factor across trials allowed us to distinguish trials in which target were updated at the same side of the body midline and those in which the target shifted to the opposite side.

Self-motion processing was reflected in a reduction of alpha band power in central parietal areas, extending laterally to the parietal-temporal areas during peak velocity and deceleration. These results nicely complement the recent findings of Gale et al. (2015) who showed a similar alpha-band suppression in these regions during body rotations. Because the motion was passively induced in the present study, we consider the alpha power modulations primarily due to vestibular processing, or the subsequent self-motion estimate derived from it (Seemungal et al., 2008). Because subjects had to maintain fixation of a body-fixed fixation point during the motion, it is also possible that the suppression of the vestibular ocular reflex (VOR), which is likely a top-down effect, originated from this activity (Ahmad et al., 2014).

Target encoding and maintenance was reflected by (contra)lateralized alpha band activity, consistent with the location of the presented and remembered target relative to midline and gaze. While we found an alpha reduction contralateral to the visually presented target, keeping it in memory gradually turned this reduction into an increase. This seems at odds with results from a previous study, using a similar paradigm, where visual and remembered targets were both reflected by a contralateral alpha *reduction* (Gutteling et al., 2015). What could explain this discrepancy? Increases in alpha power are generally considered as inhibitory while decreases are regarded as increased processing or sensitivity (Jensen and Mazaheri, 2010). Following this notion, when anticipating a visual stimulus, alpha band power generally decreases in contralateral (parieto-)occipital areas (Sauseng et al., 2005; Yamagishi et al., 2005; Thut et al., 2006). Increases are typically observed contralateral to an irrelevant or distracting spatial location (Worden et al., 2000; Kelly et al., 2006; Rihs et al., 2007). Alpha increases have also been observed during retention of working memory items (Jensen et al., 2002; Scheeringa et al., 2009), especially during sustained periods (Rihs et al., 2009), suggesting a role beyond simply inhibiting the unattended space. In fact, it has been proposed that alpha band increases could be involved in memorizing and prioritize remembered spatial items (Jensen et al., 2012). Within this context, it could be argued that task-dependencies explain the differences between the present study and our previous study. Overtly matching the location of a preserved target location, reflected by an alpha increase, was the method of establishing the remembered location in the present study. In the previous study we probed the remembered target location psychophysically, using a briefly flashed probe in a 2AFC task. It is possible that the probe location was anticipated, resulting in an alpha reduction.

Spatial updating processes were tested using the world-fixed condition, requiring the internal updating of target representations to compensate for the intervening body motion. By using variable displacement lengths and per trial responses, we inferred the updated target position per trial. As spatial updating under these conditions is predominantly gaze-fixed (Clemens et al., 2012; Gutteling et al., 2015), remembered targets that virtually cross the body/gaze midline should have their internal representations cross hemispheres. By distinguishing trials in which targets are updated across the midline (‘across’ update) and in which the target remained at the same side (‘within’ update), we characterized interhemispheric and intrahemispheric updating, respectively. Parieto-occipital alpha increases cross hemispheres when the target is updated *across* the midline, but not when the target remains on the same side relative to the midline. We further found that the initial reduction due to target presentation in the contralateral hemisphere evolves over time to an alpha increase contralateral to the updated target location. The pattern evoked by within updating is highly similar to the pattern evoked the body-fixed condition, where no updating was required.

The present results are consistent with our previous study (Gutteling et al., 2015), showing hemispheric shifts of target representations. Moreover, we found, as in our previous study, that the difference between alpha power before and after the motion correlated with the updating gain, i.e., the larger the difference, the higher the gain.

## Conclusion

We were able to disentangle the spectral signatures of self-motion estimation, target coding and spatial updating. Our results show that parietal cortex is involved in both target processing and self-motion estimation, and their selective integration to update world-fixed target or maintain body-fixed target locations during the body motion.

## Author Contributions

TG and WM designed research. TG performed research and analyzed the data. TG and WM interpreted the results and wrote the manuscript.

## Conflict of Interest Statement

The authors declare that the research was conducted in the absence of any commercial or financial relationships that could be construed as a potential conflict of interest.
